# Effects of transtheoretical model of change-based interventions on physical activity among older adults: a systematic review of randomised controlled and non-randomised controlled trials

**DOI:** 10.1186/s11556-025-00391-w

**Published:** 2025-12-02

**Authors:** Henrietta O. Fawole, Serena A. Itua, Opeyemi A. Idowu, Israel I. Adandom, Francis O. Kolawole, Tolulope Adeniji, Chigozie O. Obaseki, Olusola M. Oni, Olayinka Akinrolie

**Affiliations:** 1 University of Benin, Benin City, Nigeria; 2 Redeemer’s University, Ede, Nigeria; 3 University of Alabama, Tuscaloosa, United States; 4 Oxleas NHS Foundation Trust, Dartford, United Kingdom; 5 University of Benin Teaching Hospital, Benin City, Nigeria; 6 Buckinghamshire New University, High Wycombe, United Kingdom; 7 University of Manitoba, Winnipeg, Canada

**Keywords:** Transtheoretical model of change, Stages of change, Physical activity, Self-efficacy, Older adults

## Abstract

**Background:**

Physical inactivity in older adults is a major public health concern associated with numerous non-communicable chronic conditions. Several behaviour theories have been advanced to address the issue of physical inactivity including Transtheoretical Model (TTM) of Change among older adults. The study aimed to primarily assess the cumulative effect of TTM-based interventions on physical activity and secondarily on self-efficacy among older adults.

**Methods:**

A systematic search of electronic databases (including Cochrane Library, AgeLine, Medline, Scopus, PsycINFO, and Web of Science Core Collections) was searched from inception to August 2024. Inclusion criteria comprised studies investigating TTM-based interventions on PA in individuals aged 60 and above, randomised controlled trials (RCTs) and non-RCTs. Risk of bias was assessed using Cochrane Collaboration’s tool for RCTs while ROBIN-I was used for non-RCTs. The Grading of Recommendations Assessment, Development and Evaluation was used to evaluate the certainty of the evidence. Study findings were narratively synthesised in line with the Synthesis Without Meta-analysis framework.

**Results:**

Three studies (two RCTs and one non-RCT) met the inclusion criteria, comprising 1,474 participants (65–89 years; 71% females). TTM interventions showed low certainty of evidence of no significant effects on physical activity or self-efficacy for the RCTs. In contrast, the non-RCT showed very low-certainty evidence for the significant effects of TTM on physical activity among participants in the under-maintenance and maintenance stages, with long-term benefits limited only to those already in the maintenance stage. For self-efficacy, there was very low certainty of evidence for the significant effects of TTM only among participants in the under-maintenance stage.

**Conclusion:**

This review highlights the limited, inconsistent and low level of evidence of the effects of TTM-based interventions in promoting physical activity among older adults. Whilst for self-efficacy, there is limited, mixed and low to very low level of evidence for the beneficial effects of TTM interventions. More RCTs are needed to identify the most effective components of the TTM and understand the impact of different intervention delivery methods (e.g., digital versus face-to-face) for physical activity promotion in the older adult population.

**Supplementary Information:**

The online version contains supplementary material available at 10.1186/s11556-025-00391-w.

## Background

According to the United Nations [1], ‘an older adult is defined as a person who is over the age of 60’ whilst the World Health Organization [2], refers to older adults as individuals aged 65 years and above’. The global demographic landscape is undergoing a significant shift, with the population of older adult aged 60 years and older experiencing a rapid increase [2], particularly in the low and middle income countries [2, 3]. This trend is expected to double from the 2015 estimate of 12% to 22% by 2050, necessitating the realistic shift for inclusion of those aged 60 years and above [1, 2]. Alongside this demographic shift, there is a corresponding rise in the prevalence of non-communicable chronic diseases, including cardiovascular diseases, cancers, chronic respiratory diseases, and diabetes and increased mortality rates [4].

Physical inactivity emerges as a critical factor contributing to the development of various chronic health conditions, making it a major public health concern [5, 6]. The prevalence of physical inactivity is substantial among older adults (≥ 60 years) and has been reported to be approximately 43.5% globally in 2022 [6]. Conversely, regular and adequate physical activity has been associated with reduced risk of diseases, rate of falls, and dementia among older adults [7]. The World Health Organization recommends that older adults engage in at least 150 min of moderate-vigorous intensity physical activity per week [8]. Interventions targeting behaviour change are important in promoting physical activity, and various theoretical frameworks and models have been employed. Among these, the Transtheoretical Model (TTM) of Change, proposed by Prochaska and Diclemente (1983) [9], emerges as a promising approach.

TTM is a comprehensive model that argues that change is not a discrete event but a process that takes time to occur [9]. The model posits that behaviour change is a dynamic process with a temporal dimension [10]. An individual moves through a sequence of change from a point with no intention to change behaviour to maintaining change [10]. The TTM was initially developed through evidence from smoking cessation studies [11], but its application has since expanded to other behavioural change including physical activity. The model is considered to be one of the most popular models used to understand stages of change in physical activity behaviour [12]. TTM premised that change occurs through six stages: pre-contemplation, contemplation, preparation, action, maintenance, and termination stages. Prochaska et al. defined pre-contemplation as a stage where individual is not thinking about making change or motivated to change [13]. The contemplation stage is when the individual begins to think about changing their behaviour and considers the pros and cons of change [13]. When the individual begins to think about taking action in the immediate future is called preparation stage [13]. In the action stage, an individual has made change to their behaviour and working to prevent relapse is called maintenance stage [13]. Finally, the termination stage is when the individual has developed sufficient self-efficacy and are certain that they will not return to their previous behaviour [13].

Other constructs of this model include processes of change (strategies and techniques that individual employ to modify their behaviour in order to progress along the stages of change), decisional balance (an individual’s evaluation regarding the cons and pros of engaging in a behaviour), and self-efficacy (one’s belief in one’s ability to perform specific behaviours in specific situations) [10]. Among older adults, higher self-efficacy is associated with greater adoption and maintenance of physical activity, as it enhances confidence in overcoming age-related barriers [14]. Equally, self-efficacy mediates the relationship between processes of change and stages of change [15]. However, the stages of change have received the most attention and are widely studied. TTM has been shown to be effective in promoting physical activity in the general adult population [12, 16], however, there is limited evidence on the cumulative effects of TTM on physical activity among older adults. Previous systematic reviews have focused on the relationship between TTM and physical activity in either older adults [17], or the general adult population [18], but not specially on the effect of TTM based intervention on physical activity promotion among older adults. This is particularly significant for the older adult population, where various age-related physiological, psychological, and social factors can affect physical activity behaviors and responses to interventions [19]. Further, considering the benefits of promoting physical activity in this population, it is vital to investigate the potential causal mechanisms behind the observed effects of TTM-based interventions on physical activity. Therefore, this systematic review aimed to primarily determine the effect of TTM-based interventions on physical activity outcomes among older adults and to synthesise the current evidence. Physical activity was considered the primary outcome, while self-efficacy was a secondary outcome. Specifically, we sought to answer the following research questions: (i) what is the effect of TTM-based interventions on physical activity among older adults? and (ii) what is the effect of TTM-based interventions on self-efficacy among older adults?

## Method

### Review

This review was carried out in accordance with the Cochrane Handbook for Systematic Review of Interventions [20]. The reporting of this systematic review followed the Preferred Reporting Items for Systematic Review and Meta-analysis (PRISMA) [21] and the Synthesis without Meta-analysis (SWiM) Extension guidelines [22]. A review protocol was developed and registered in International prospective register of systematic reviews (PROSPERO) database, with registration ID: CRD42023442344.

### Study criteria and selection

#### Population, intervention, comparators, outcomes, study types and settings

The PICOS (Population, Intervention, Comparisons, Outcomes, Study design) framework was used to structure the inclusion and exclusion criteria for study selection ensuring that the review process is guided by the research question [23].

Population - Older adults aged 60 and above, a mixed population reporting subgroup analysis on older adults aged 60 and above.

Intervention - TTM-based interventions.

Comparator – Standard care, placebo or no intervention.

Outcomes – The primary outcome of this review was physical activity, measured either subjectively or objectively and the secondary was self-efficacy.

Study design - randomized controlled trials or non-randomized control trials.

Settings: All settings.

### Inclusion criteria

Studies were included if they met the following criteria: (a) a study population of older adults aged 60 and above, a mixed population reporting subgroup analysis on older adults aged 60 and above; b) utilized TTM-based interventions on physical activity (c) assessed physical activity either subjectively or objectively and/or assessed self-efficacy (d) any trial design - randomized controlled trials or non-randomized control trials (e) study published in English Language. Our primary and secondary outcomes were physical activity and self-efficacy respectively.

### Exclusion criteria

Studies were excluded if they were thesis reports, protocols, and abstracts or studies not published in the English Language. Studies that used a combination of other theories and TTM-based interventions on physical activity were excluded. Studies of older adults with dementia, cognitive impairments and neurodegenerative disorders and animal studies were also excluded.

### Search strategy

The following electronic databases were searched from inception of each database to August 2024:

Cochrane library (Ovid) – 1995 to August 2024.

AgeLine (EBSCO) – 1978 to August 2024.

Medline (Ovid) – 1946 to August 2024.

Scopus (Elsevier) – 1788 to August 2024.

PsycINFO (Ovid) – 1806 to August 2024.

Web of Science core collections (Clarivate) – 1900 to August 2024.

Keywords used for the search included “Older adults” OR “Seniors” OR “Elderly” AND “Transtheoretical model of change” OR “TTM” OR “Stages of change” AND “Physical activity” OR “walking” OR “walk^*^” OR “Exercise”. The search strategy was reviewed by a team of experts in physical activity and gerontology research and systematic review methodology. The reference lists of all the included studies were hand-searched for additional eligible studies. The search strategy for all the databases is attached in the supplementary material (S1).

### Data management

All the articles found from the databases were exported into the COVIDENCE (www.covidence.org), a systematic review software where duplicates were removed. After which, the rest of the articles were screened based on inclusion and exclusion criteria.

### Study selection

Two reviewers SAI and FOK independently screened titles and abstracts. Two reviewers SAI and FOK independently screened titles and abstracts. Thereafter, both reviewers screened eligible full texts against the inclusion criteria. In cases of disagreement, HOF and OA were consulted for the final decision.

### Data extraction

Two reviewers (SAI and HOF) extracted the following information independently from all included studies using a pre-piloted data extraction form: Name of author(s), year of publication, country, study design, population characteristics, TTM intervention description, control intervention description, mode of delivery, duration and frequency of the intervention, outcome measurement tools, findings, strengths and limitation. Discrepancies were resolved through discussion.

### Risk of bias

Two reviewers (SAI and FOK) independently assessed the risk of bias in each trial using the Cochrane Collaboration risk of bias-2 tool for randomised controlled trials (RCTs) (ROB-2) [24]. This tool assesses the risk of bias such as randomisation process, deviations from intended interventions, missing outcome data, measurement of outcome and selection of the reported result. Each risk of bias was rated as “high risk”, “low risk” and “some concerns”. The Cochrane ROBINS-I is a commonly recommended tool for non-randomized clinical experiments [25]. ROBINS-I tool was used to assess the risk of bias of included non-randomized studies. Risk of bias was classified as low, moderate, serious or critical based on bias in these seven domains: confounding, participant selection, intervention, measurement, deviation from intended intervention, missing data, outcome measurement and selection of reported result. A study was considered to have a low risk of bias if all the domains were considered as low; moderate if the domains were ranked as low or moderate; serious if at least one domain was considered serious; and critical, if at least one domain was considered critical [25]. In cases of disagreement, a third reviewer (HOF) was consulted. The risk of bias tool was pilot-tested independently by the two reviewers using one of the RCT studies to ensure consistency and familiarisation with the process.

### Dealing with missing data

Where there was missing data, the authors attempted to obtain relevant missing data from authors of the included trials. In addition, we evaluated important numerical data such as screened, eligible and randomly assigned participants, as well as intention to treat and per protocol populations. Further, we investigated attrition (drop-outs, losses to follow ups, and withdrawal).

### Data synthesis

Data of the included studies was synthesised narratively using the SWiM guidelines [22]. Further, the characteristics of the included studies were summarised in a tabular form. Due to the limited number of studies and heterogeneity in study design, a meta-analysis was not conducted, and results were presented using a narrative synthesis. In addition, certainty of evidence was assessed using Grading of Recommendations, Assessments, Development and Evaluation (GRADE) framework [26]. Evidence was rated as high, moderate, low and very low considering factors such as risk of bias, inconsistency, indirectness, imprecision and publication bias [26]. This is consistent with established guidance indicating that GRADE can be applied even in the absence of pooled effect estimates [27]. This approach is often adopted in clinical studies [28].

## Results

### Study characteristics

**Fig. 1:**
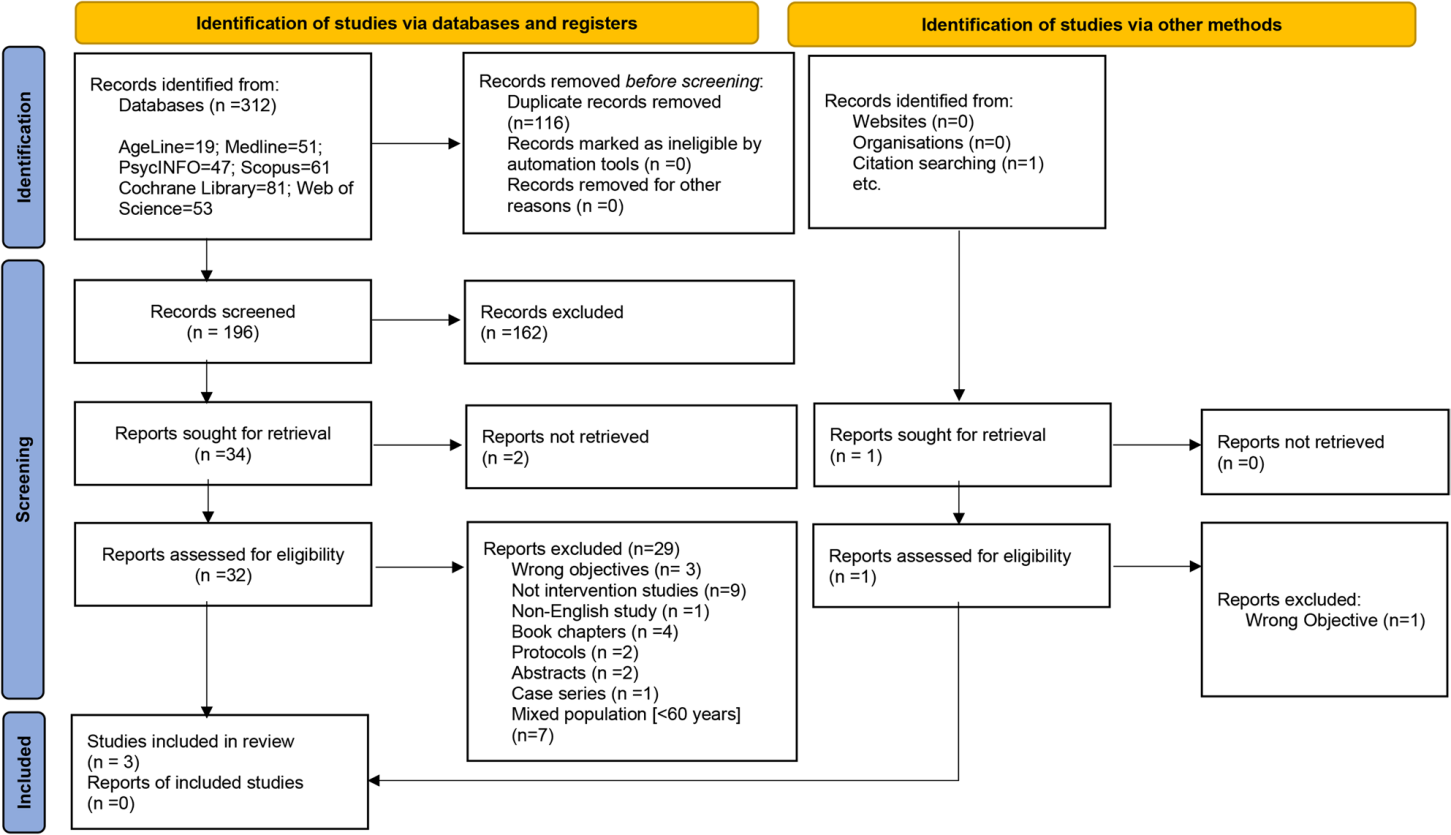
PRISMA flow diagram of the study selection

The search resulted into 312 articles and only three studies were included in this review [29–31]. The PRISMA flow diagram shows the number of excluded studies and the reasons for exclusion (Fig. 1).

The total number of participants in the included studies is 1274 and the sample sizes ranged from 30 to 1274 participants [29–31]. Participants were recruited from an outpatient clinic [31] and community settings [29, 30]. Two of the three included studies were parallel RCTs [29, 31] while one was non-RCT [30]. The studies were conducted in three different countries: Germany [31], United States [29] and Taiwan [30]. Physical activity was measured using a 7-day activity diary [31], Yale Physical Activity Survey (YPAS) [29], and Physical Activity Scale for the Elderly (PASE) [30]. Two of the three included studies assessed self-efficacy. Greaney et al. [29] used the six-item exercise self-efficacy scale while Hsu et al. [30] used self-efficacy for exercise scale.

### Intervention description

The TTM-based interventions were delivered via counselling sessions [31], print materials and coaching calls [29], or group exercises tailored based on individual’s exercise stages of change - pre-maintenance stage or maintenance stage [30]. Basler et al. [31] delivered ten counselling sessions which was conducted in-person for a duration of 10 min per session over a period of 5 weeks [31]. On the other hand, Greaney et al. [29] utilised print materials, and manual were administered at the start of the interventions while newsletters were delivered on a monthly basis except months 4, 8 and 12. In addition to the print materials, coaching calls were delivered in 3 sessions for a duration of 15 min per session. The group exercises for people under the maintenance stage and people in the maintenance stage took place every Saturday for 60 min for 24 weeks [30]. The studies utilised physiotherapists, a counsellor, and an athletics fitness instructor to deliver the interventions to the participants. Detailed descriptions of the interventions are presented in Table 1.

**Table 1:** Characteristics of all included studies

Authors, Year/Country; Study design	Description of TTM intervention	Control intervention description	Mode of delivery (duration, frequency/intervention provider); Settings	Outcome measurement tool used	Findings, Strengths and Limitations
Basler et al., 2007 [31]/Germany Parallel RCT	The patients individually attended a standardised counselling procedure of 10 min duration prior to every physiotherapy treatment. The programme addressed readiness for change and integrated some relevant processes of change such as consciousness raising. The strategies were aimed at increasing self-efficacy and positively influencing decisional balance. The counselling took into consideration the individual’s stage of change that was determined during the initial assessment. In addition, patients received patient-tailored 20 min treatment session and a standardised treatment manual focused on activities of daily living. Treatment was targeted at improving trunk and lower limb muscle length, strength, flexibility and coordination.	The control group received a 20-minute tailored treatment session, followed by a standardised treatment manual focused on activities of daily living. Treatment was targeted at improving trunk and lower limb muscle length, strength, flexibility and coordination. In addition, patients received a placebo ultrasound therapy with an inactive device for a duration of 10 min.	**Mode of delivery** 5 weeks, 10 sessions (10 min per session for the standardised counselling based on stage of change and 20 min for the tailored treatment session)/Physiotherapists **Settings** Outpatient clinic	PA: 7-day activity diary Self-efficacy: Not assessed	**Findings**: Both TTM intervention and control conferred similar improvement on PA. However, the time spent on PA for the intervention group was slightly greater than that of the control group. **Strengths**: (1)This study was a randomised intervention study with a control group and follow-up period of 6 months. (2) Inclusion of a placebo treatment may have prevented an occurrence of non-specific treatment effects in the control group. (3) The counselling which was based on TTM intervention took into consideration the participant’s baseline stage of change. **Limitations**: The sample size was not large enough to determine whether participants in the pre-action stages benefited more from TTM intervention than those in the action stages.
Greaney et al., 2008 [29]/USA Parallel RCT	The intervention was developed based on TTM and it consisted of print materials (manuals at the start of intervention and newsletters on a monthly basis except for months 4, 8 and 12 when they received an expert system report) and three 15 min coaching calls encouraging participants to engage in aerobic exercise of moderate to vigorous intensity for 3 to 5 days per week for a minimum of 20 min; flexibility exercises for at least 2 days per week; and muscle-strengthening exercises for 2 to 3 days per week designed for transition through the different stages of change.	The control group received either a manual about fruit and vegetable consumption or fall-prevention manual.	**Mode of delivery** 12 months, 3 sessions of 15-minute calls/Trained counsellors. **Settings** Community	PA: Yale physical activity survey (YPAS). Self-efficacy: Six-item exercise self-efficacy scale	**Findings**: The study showed no difference between the intervention and comparison group in PA and self-efficacy scores after 12 or 24 months. **Strengths**: (1) This study was a randomised intervention study with a control group. (2) The intervention was based on TTM which encouraged participants to engage in aerobic exercises, flexibility exercises and muscle-strengthening exercises. (3) This study had a follow up of 12-month intervention period, and 12 months post intervention, during which no intervention was delivered. **Limitations**: (1) A major limitation was the possibility of sampling bias (volunteer bias), where those in the pre-action stages may have been less willing to volunteer as study participants while people in maintenance may have been more likely to do so. (2) There was also the possibility of drop-out bias because individuals who were sedentary and with lower self-efficacy at baseline were more likely to withdraw than individuals who regularly exercised. (3) PA and self-efficacy were measured by self-report, which can reduce the sensitivity of the instruments as a result of report and response biases.
Hsu et al., 2022 [30]/Taiwan A single-arm clinical trial	The intervention comprised two phases: a structured class (1st to 12th week) and an autonomous class (12th to 24th week). Participants were divided based on their baseline exercise stage of change into maintenance stage and under maintenance stage. Further, in preparation for the autonomous class phase, subjects were evenly allocated into two groups (A and B) based on their exercise behaviour pattern (stage of change, self-efficacy, PASE, Outcome expectations) while ensuring an equal representation of the stage of change groups. The subjects participated in a weekly, 60-minute mini-ball group fitness class, leaving 10 min for participants to discuss their exercise experience and give mutual emotional support. Additionally, the fitness class instructor provided relevant information about opportunities for fitness within the community. During the 1 st to 4th weeks, the instructor led subjects to exercise and mentored the team leaders of groups A and B for future leadership. The group leaders of each group took over the leadership of the exercise sessions in their respective groups during the 5th to 8th week while the instructor provided support and counsel. During the 9th to 12th week, the deputy leader for each group led the exercise sessions while the group leaders provided guidance and counselling. In addition to the group activities, each group was encouraged to organise additional one or two classes per week as deemed convenient by group members. During the autonomous class phase, a line group was established. Each group (A and B) invited her members to engage in the same mini-ball exercise sessions for an additional 12 weeks. Video footage of exercise sessions was presented to get a reward. The test scores of the 24th week was compared with the 12th week to determine progress. The progress scores and line scores were added to the line group exercise scores and the higher total scores won. Each member of the group was then given a $100 gift voucher. Feedback in form of personal progress report and explanations for progress or shortcomings were made available to subjects.	No control group	**Mode of delivery** 24 weeks, every Saturday (for 60 min)/Trained Athletics and Fitness Instructor. **Settings** Community care centre	PA: Physical activity scale for the elderly (PASE) Self-efficacy: Self-efficacy for exercise scale	**Findings**: In the short term (12 weeks), TTM intervention model successfully improved PA for individuals in the maintenance and under maintenance stages. However, sustained benefits (24 weeks) were noted for those in the maintenance stage. For exercise self-efficacy, people under the maintenance stage had beneficial improvement in the short term relative to those in the maintenance stage. **Strengths**: (1) The study was an intervention study based on TTM with a 12 and 24 weeks follow up period. (2) The study self-developed and designed fitness exercises that were suitable for the elderly. (3) The intervention provider (trained athletics and fitness instructor) strengthened the proficiency of the team leaders in the group exercises in order to transfer his role for them to take over. **Limitations**: (1) The study was not a randomised controlled trial. (2) The small sample size makes it unable to generalize the results of the survey to the population as a whole. (3) The study adopted self-reported questionnaires for the PA and self-efficacy.

*TTM* Transtheoretical model of change, *IG * intervention group, *CG* control group, *PASE* Physical activity scale for the elderly, *PA* Physical activity,* n* sample size, *YPAS* Yale physical activity scale

#### Risk of bias

**Fig. 2:**
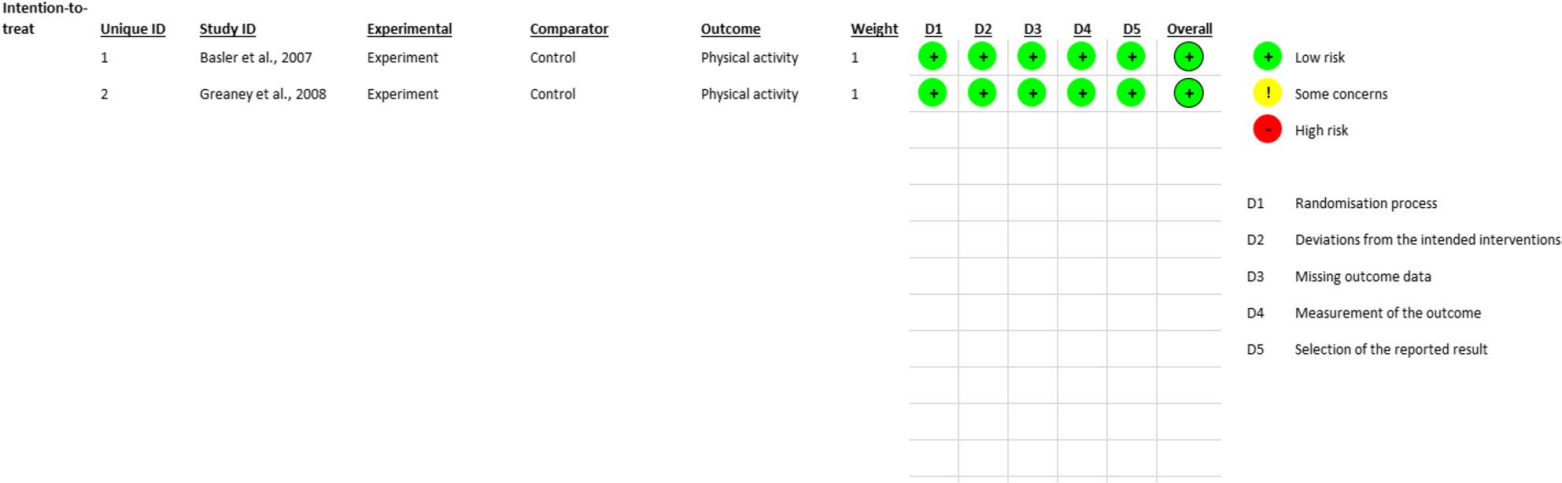
Cochrane risk of bias for parallel randomized controlled trials (RCTs)

The two RCT studies were rated to have high quality as a result of the low risk of bias [29, 31] (Fig. 2). Randomisation process, deviation from the intended interventions, missing outcome data, measurement of the outcome and the selection of the reported result were assessed and rated as having low risk of bias. The non-RCT study using the ROBIN-I had an overall serious RoB (Table 2). Bias due to confounding and bias in measurement of outcomes were assessed to have serious risk of bias.

**Table 2:** ROBIN-I risk of bias for non-RCT study

Authors	Bias due to cofounding	Bias due to selection of participants into the study	Bias due to classification of intervention	Bias due to deviations from intended intervention	Bias due to missing data	Bias in measurement of outcomes	Bias in selection of the reported result	Risk of Bias status
Hsu et al., 2022 [30]	Serious	Low	Low	Low	Low	Serious	Low	Serious RoB

*RoB * Risk of Bias

## Narrative synthesis

### Effects of TTM-based interventions on PA

#### RCT

Basler et al. [31] showed that compared to the control group, TTM group showed no significant increase in physical activity level from baseline to follow-up. Similarly, Greaney et al. [29] found no significant differences between intervention and control groups on physical activity level (Table 3; Supplementary material S2). The certainty of evidence for physical activity based on GRADE approach was downgraded to low (Table 4), primarily due to serious inconsistency (heterogeneity across the two studies) and serious imprecision.

**Table 3:** Effects of TTM interventions on physical activity among older adults

		Interventions		
Author/Year/Type of Study Outcome: measure	Sample size; Sex (m/f); Dropout. Age mean (SD) or Age Range (IQR)	Intervention	Control	Effectiveness of TTM vs. Control
Basler et al. (2007) [31]/RCT *PA-7-day activity diary*	*n* = 170; IG = 86 (m/f) (32/54), CG = 84 (m/f) (29/55) IG = 75, CG = 72; dropout = 23 Age mean (SD) = 70.3(4.4), IG = 70.09(4.19), CG = 70.56(4.55) Age r**ange: 65–84 years**	**Baseline**	**Baseline**	
		15.98 (21.1)	14.11 (15.5)	No statistically significant difference
		**Post (6/7 weeks)**	**Post (6/7 weeks)**	
		29.24 (14.6)	24.7 (16.3)	
		**Change**	**Change**	
		13.26 (−6.5)	10.59 (0.8)	
		**Follow-up (6-months)**	**Follow-up (6-months)**	
		29.63 (24.2)	25.3 (19.7)	
		**Change**	**Change**	
		13.65 (9.6)	11.19 (4.2)	
Greaney et al. (2008) [29]/RCT *PA: Yale Physical Activity Survey (YPAS)*	*n* = 1274 IG(m/f) = 470(128/342); CG(m/f) = 496(146/348); dropout = 308 Age mean (SD): IG = 75.2(6.7), CG = 74.7(6.6)	**Baseline**	**Baseline**	
		46 (1.4)	46 (1.3)	No statistically significant difference
		**Post (12-months)**	**Post (12-months)**	
		46 (1.2)	47 (1.1)	
		**Change**	**Change**	
		0 (−0.2)	1 (−0.2)	
		**Follow-up (24-months)**	**Follow up (24-months)**	
		47 (1.3)	47 (1.2)	
		**Change**	**Change**	
		1 (−0.1)	0.11 (0.01)	
Hsu et al. (2022) [30]/Single arm clinical trial (non-RCT)^a^ *PA: Physical Activity Scale for Elderly*	*n* = 30; MPb = 15 (m/f) = 4/11 MP = 13; (m/f) = 3/10 dropout = 2 Age mean (SD) = 68.8(4.1); Age range: 66–89 years.	**Baseline**	**Baseline**	
		MP_b =_122.70 (56.73)	N/A	TTM improved PA in both groups
		MP = 230.17 (55.60)	N/A	
		**Post (12-weeks)**	**Post (12-weeks)**	
		MP_b_ = 208.14 (52.29)	N/A	
		MP = 264.77 (93.52)	N/A	
		**Change**	**Change**	
		MP_b_ = 85.44 (−4.44)	N/A	
		MP = 34.6 (37.92)	N/A	
		**Follow-up (24-weeks)**	**Follow-up (24-weeks)**	
		MP_b_ = 177.92 (45.39)	N/A	
		MP = 284.27 (154.87)	N/A	
		**Change**	**Change**	
		MP_b_ = 55.22 (−11.34)	N/A	
		MP = 54.1 (99.27)	N/A	

*PA* Physical activity, ^a^ This study *had two intervention groups, MP*_b_ *under maintenance stage*, *MP maintenance stage*; *N/A Not applicable,* *RCT Randomised Controlled Trial, * *TTM Transtheoretical model of based change*

**Table 4:** GRADE certainty of evidence results

No of participants (studies)	Design	Risk of bias	Inconsistency	Indirectness	Imprecision	Publication bias	Certainty
Physical activity (PA)							
1113 (2)	RCT	Not Serious	Serious^a^	Not Serious	Serious^b^	Not likely	⊕⊕◯◯ Lowᵃᵇ
28 (1)	Non-RCT	Serious^c^	Serious^d^	Not Serious	Serious^d^	None	⊕◯◯◯ Very Low ^cd^
Self-efficacy							
966 (1)	RCT	Not Serious	Not Serious	Not Serious	Very Serious^b^	None	⊕⊕◯◯ Lowᵇ
28 (1)	Non-RCT	Serious^c^	Serious^d^	Not Serious	Serious^d^	None	⊕◯◯◯ Very Low ^cd^

a. _Serious inconsistency due to heterogeneity in effect sizes across studies_

b. _Serious/Very serious imprecision due to wide confidence intervals crossing the line of no effect_

c. _Serious risk of bias due to bias to confounding and bias in measurement of outcomes_

d. _Serious inconsistency/imprecision as the non-RCT is a pre-post study, and we have only one eligible non-RCT to include_

#### Non-RCT

Hsu et al. [30] showed that TTM intervention successfully improved PA for individuals in both the maintenance stage and under-maintenance stage in the short term (12 weeks). However, sustained benefit was only noted for those in the maintenance stage [30] (Table 3). The certainty of evidence for physical activity based on GRADE approach was downgraded to very low (Table 4), primarily due to serious risk of bias, imprecisions and inconsistency.

### Effects of TTM-based interventions on self-efficacy

#### RCT

The RCT study found no significant differences within and between groups for self-efficacy (Table 5; Supplementary material S2) [29]. The certainty of the evidence was downgraded to low due to very serious imprecision (Table 4).

**Table 5:** Effects of TTM on self-efficacy among older adults

		Interventions		
Author/Year/Type of Study Outcome	Sample size; Sex (m/f); Dropout. Age mean (SD) or Age Range (IQR)	Intervention	Control	Effectiveness of TTM vs. control
Greaney et al. (2008) [29]/RCT *Self-efficacy: Six item exercise self-efficacy scale*	*n* = 1274 IG(m/f) = 470(128/342); CG(m/f) = 496(146/348); dropout = 308 Age mean (SD): IG = 75.2(6.7), CG = 74.7(6.6)	Baseline	Baseline	
		3.41 (0.04)	3.37 (0.04)	No statistically significant differences
		Post (12-months)	Post (12-months)	
		3.50 (0.05)	3.41 (0.04)	
		Change	Change	
		0.09 (0.01)	0.04 (0.00)	
		Follow-up (24-months)	Follow-up (24-months)	
		3.52 (0.05)	3.41 (0.05)	
		Change	Change	
		0.11 (0.01)	0.04 (0.01)	
Hsu et al. (2022) [30]/non-RCT ^a^ *Self-efficacy: Self Efficacy for Exercise Scale*	*n* = 30; MPb = 15 (m/f) = 4/11 MP = 13; (m/f) = 3/10 dropout = 2 Age mean (SD) = 68.8(4.1); Age range: 66–89 years.	Baseline	Baseline	
		MP_b_ = 5.52 (2.34)	N/A	TTM improved self-efficacy in both groups
		MP = 8.32 (1.49)	N/A	
		Post (12-weeks)	Post (12-weeks)	
		MP_b_ = 8.06 (1.59)	N/A	
		MP = 8.11 (1.48)	N/A	
		Change	Change	
		MP_b_ = 2.54 (−0.75)	N/A	
		MP = −0.21 (−0.01)	N/A	
		Follow-up (24-weeks)	Follow-up (24-weeks)	
		MP_b_ =6.84 (1.99)	N/A	
		MP = 8.34 (1.28)	N/A	
		Change	Change	
		MP_b_ =1.32 (−0.35)	N/A	
		MP = 0.02 (−0.21)	N/A	

*PA Physical activity; a This study had two intervention groups, MPb under maintenance stage*,* MP maintenance stage, N/A Not applicable, RCT Randomised Controlled Trial, TTM Transtheoretical model of based change*

#### Non-RCT

The non-RCT study showed that people under the maintenance stage showed beneficial improvement for self-efficacy in the short term (12 weeks) relative to individuals in the maintenance stage [30] (Table 5). The certainty of evidence for self-efficacy based on GRADE approach was downgraded to very low (Table 4), primarily due to serious risk of bias, imprecisions and inconsistency.

## Discussion

This systematic review examined the effects of TTM-based interventions in promoting physical activity and enhancing self-efficacy among older adults aged 60 years and above. Only three studies met the eligibility criteria, and the evidence was limited and inconsistent. Two randomised controlled trials (RCTs) found low certainty of no significant effects of TTM on physical activity [29, 31]. Specifically, Basler et al. [31] reported that the addition of TTM to usual care did not improve physical activity beyond what was achieved with usual care alone. Similarly, Greaney et al. [29] found no differences in physical activity levels between intervention and control groups. This null findings may partly reflect the inclusion of high performing older adults already in the action or maintenance stages at baseline [29, 31]. In contrast, the non-RCT by Hsu et al. [30] suggested that TTM-based interventions led to short-term improvements in physical activity among participants in the maintenance and under-maintenance stages. However, the long-term benefit was confined to those already in the maintenance stage, with very low certainty of evidence. This finding highlights the importance of tailoring interventions to participants’ stages of change, as their readiness to adopt and sustain physical activity behaviours may influence intervention outcomes.

A previous systematic review by Jimenez-Zazo et al. [17] included cross-sectional and quasi-experimental studies examining associations between TTM and physical activity in older adults. Unlike our review, which focussed strictly on intervention effects of TTM on physical activity, they found only one intervention [32]. This study was not included in our current review because it focused on functional fitness rather than physical activity outcomes, raising questions about its inclusion in the earlier review. Together, these findings underscore the paucity of studies on TTM-based interventions on physical activity in the older adults population.

Further, our finding of limited and inconsistent evidence on the effect of TTM on physical activity in older adults contrasts with those of Kleis et al. [16], who reported mixed evidence of TTM effectiveness in healthy adults, with some studies showing improvements (e.g., Kolt et al. [33]; Petrella et al. [34]), and others finding no differences between intervention and control groups (e.g., Blissmer & McAuley [35]; Marshall et al. [36]). Variability across the two reviews likely reflects differences in populations, outcomes, study designs, and delivery modes.

The secondary outcome, self-efficacy, also showed limited, inconsistent results and low to very low level of evidence. Greaney et al. [29] found no significant improvements in self-efficacy among intervention participants, suggesting a limited impact of TTM in this context. Conversely, Hsu et al. [30] reported short-term improvements in self-efficacy among participants in the under-maintenance stage. Although intervention studies remain scarce, observational research has shown positive associations between self-efficacy and advanced stages of change among older adults [17]. As self-efficacy is critical transitions from contemplation to maintenance stages, stage-matched strategies that address constructs such as decisional balance and temptation, alongside self-efficacy, may enhance [37].

Although our review found inconsistent and low-certainty evidence of no beneficial effects of TTM-based interventions on physical activity in older adults, this does not contradict the well-established benefits of physical activity. Ample evidence links higher levels of physical activity and structured exercise with reduced mortality, better function, and improved psychological health in older adults, whilst sedentary behaviour is consistently linked to adverse health outcomes [38, 39]. Exercise also enhances self-efficacy, largely through mastery experiences and improved competence [40, 41]. The discrepancy between the substantial benefits of physical activity and the limited effects of TTM-based interventions most likely reflects implementation challenges such as incomplete application of TTM constructs, insufficient intervention intensity, and varied context or poor adherence. Future research should therefore focus on optimising TTM delivery to ensure that interventions achieve the behavioural changes required to realise the well-documented health and self-efficacy benefits of physical activity.

Regarding the incomplete application of TTM constructs during intervention, the included studies used different components of TTM (Basler et al. [31]: all components; Greaney et al. [29]: stages of change; Hsu et al. [30]: stages of change and self-efficacy). A meta-analysis of 33 studies suggested that incorporating at least three constructs into interventions aimed at promoting physical activity is necessary for effectiveness [42]. Yet, Basler et al. [31], which used all components, showed no significant improvement in physical activity, raising the question of whether interventions are truly theory-driven or merely inspired by TTM, as argued by Romain et al. 2018 [42] and Ntoumanis et al. [43]. Future interventions should explicitly describe how constructs are applied, to help identify the most important components, as this may support sustainability, adherence, and overall effectiveness.

Further extending this discussion, study contextual and delivery differences may also have influenced our findings. For instance, Greaney et al. [29] and Hsu et al. [30] conducted their interventions in community settings, whilst Basler et al. [31] used an outpatient clinic. However, the intervention delivery modes differed in the community-based studies [Greaney et al. [29] (telephone) and Hsu et al. [30] (face to face)]. Both face-to-face and remote interventions (e.g., telephone) have been shown to promote physical activity in older adults [44, 45], however, there appears to be a dearth of studies directly comparing their relative effectiveness. Future studies should therefore not only clarify these differences but also report implementation setting and social interaction levels [46, 48].

Some limitations of this review must be considered when interpreting our findings. First, the small numbers of studies found limits the generalisation of the findings. Second, the heterogenous TTM constructs, and varied designs used in the included studies precluded meta-analysis. Restricting to English Language publications studies may have led to the exclusion of other studies published in other languages, thus potentially introducing language bias. However, given that TTM-based interventions are not language specific, all relevant studies are expected to have been covered in English publications – thus limiting the impact of language bias on the conclusions of this review. It is also essential to consider the strength of this review which included the adherence to Cochrane Handbook for Systematic Review of Interventions, the use of Cochrane risk of bias-2 (RoB-2) for the assessment of internal validity of the RCTs and presentation of our finding according to PRISMA and SWiM guidelines. Further, our review included all studies irrespective of comorbidities, which is likely to be representative of the older adult population. In addition, assessing certainty of evidence using GRADE improved the rigour of our systematic review.

To strengthen the evidence and improve intervention outcomes, future research should prioritise adequately powered RCTs to evaluate different TTM constructs and their impact on physical activity. Detailed reporting of intervention methods, participant characteristics, and baseline readiness for change is essential for reproducibility and validity. Integration of TTM strategies into geriatric physiotherapy, coupled with mobile or wearable technologies, could provide personalised feedback and promote sustained behaviour change. Exploring the integration of complementary behaviour-change models could further enhance the effectiveness of TTM-based interventions. Given the diversity of older adult population, factors such as multimorbidity, social support, environmental constraints, use of theory, and intervention delivery must be considered to improve physical activity and self-efficacy outcomes in the older adult population [48, 49].

## Conclusion

This review highlights the limited, inconsistent and low level of evidence of the effects of TTM-based interventions in promoting physical activity among older adults. Whilst for self-efficacy, there is limited, mixed and low to very low level of evidence for the beneficial effects of TTM interventions. More RCTs with keen focus on intervention design, and contextual factors are needed to identify the most effective components of the TTM and understand the impact of different intervention delivery methods (e.g., digital versus face-to-face) for physical activity promotion in the older adult population.

## Supplementary Information

Supplementary Material 1.

## Data Availability

No datasets were generated or analysed during the current study.

## Abbreviations

TTM: Transtheoretical model
PRISMA: Preferred reporting items for systematic review and meta-analysis
PROSPERO: International prospective register of systematic reviews
RCT: Randomised controlled trial
ROB: Risk of bias
YPAS: Yale physical activity survey
PASE: Physical activity scale for the elderly
PICOS: Population, intervention, comparisons, outcome, study design
