# Supplementary material for: Effects of transtheoretical model of change-based interventions on physical activity among older adults: a systematic review of randomised controlled and non-randomised controlled trials
Source: Eur Rev Aging Phys Act. 2025 Dec 2;23:1. doi: 10.1186/s11556-025-00391-w (PMC12781522; doi:10.1186/s11556-025-00391-w)
Supplement: Supplementary file 1 — Supplementary Material 1. [file 11556_2025_391_MOESM1_ESM.docx]

# Supplementary Material

S1: Search strategies for electronic databases (First search - 15th July 2023 and updated 8^th^ August 2024).

| **Databases** | **Search Terms** |
| --- | --- |
| AgeLine (Ebscohost)  **n= 19** | S1= TI (“older adults” OR “seniors” OR “elderly”) OR AB (“older adults” OR “seniors” OR “elderly”) = **77,187**  S2= TI (“transtheoretical model of change” OR “TTM” OR “Stages of change”) OR AB (“transtheoretical model of change” OR “TTM” OR “Stages of change”) = **75**  S3= TI (“physical activity” OR “walking” OR “walk*” OR “exercise”) OR AB (“physical activity” OR “walking” OR “walk*” OR “exercise”) = **12,971**  S4= S1 AND S2 AND S3 = **24**  with limiter to academic literature = **19** |
| Medline (Ovid)  **n= 51** | 1= (‘Older adults’ or ‘Seniors’ or ‘Elderly’).mp. [mp=title, book title, abstract, original title, name of substance word, subject heading word, floating sub-heading word, keyword heading word, organism supplementary concept word, protocol supplementary concept word, rare disease supplementary concept word, unique identifier, synonyms, population supplementary concept word, anatomy supplementary concept word] = **407,938**  2= (‘Transtheoretical model of change’ or ‘TTM’ or ‘Stages of Change’).mp. [mp=titie, book title, abstract, original title, name of substance word, subject heading word, floating sub-heading word, keyword heading word, organism supplementary concept word, protocol supplementary concept word, rare disease supplementary concept word, unique identifier, synonyms, population supplementary concept word, anatomy supplementary concept word] = **4,066**  3= (‘Physical activity’ or ‘Walking’ or ‘walk’ or ‘Exercise’) mp. [mp=title, book title, abstract, original title, name of substance word, subject heading word, floating sub-heading word, keyword heading word, organism supplementary concept word, protocol supplementary concept word, rare disease supplementary concept word, unique identifier, synonyms, population supplementary concept word, anatomy supplementary concept word] = **617,861**  4= 1 and 2 and 3 = **53**  5= limit 4 to English language = **51** |
| PsycINFO (Ovid)  **n= 47** | 1= (Older adults or Seniors or Elderly).mp. [mp=title, abstract, heading word, table of contents, key concepts, original title, tests & measures, mesh word] = **131,796**  2= (Transtheoretical model of change or TTM or Stages of Change).mp. [mp=title, abstract, heading word, table of contents, key concepts, original title, tests & measures, mesh word] = **3,987**  3= (Physical activity or walking or walk* or Exercise).mp. [mp=title, abstract, heading word, table of contents, key concepts, original title, tests & measures, mesh word] = **130,079**  4= 1 and 2 and 3 = **48**  5= limit 4 to English language = **47** |
| Scopus (Elsevier)  **n= 61** | 1= TITLE-ABS-KEY (“older adults” OR “seniors” OR “elderly”) = **1,101,587**  2= TITLE-ABS-KEY (“transtheoretical model of change” OR “TTM” OR “stages of change”) = **7,918**  3= TITLE-ABS-KEY (“physical activity” OR “walking” OR “walk*” OR “exercise”) = **153,103**  4= (TITLE-ABS-KEY (“older adults” OR “seniors” OR “elderly”)) AND (TITLE-ABS-KEY (“transtheoretical model of change” OR “TTM” OR “stages of change”)) AND (TITLE-ABS-KEY (“physical activity” OR “walking” OR “walk*” OR “exercise”)) = **61**  5= (TITLE-ABS-KEY (“older adults” OR “seniors” OR “elderly”)) AND (TITLE-ABS-KEY (“transtheoretical model of change” OR “TTM” OR “stages of change”)) AND (TITLE-ABS-KEY (“physical activity” OR “walking” OR “walk*” OR “exercise”)) AND (LIMIT-TO (LANGUAGE, “English”)) = **61** |
| Web of Science core collections (Clarivate)  **n= 53** | 1= “older adults” OR “seniors” OR “elderly” (Title) OR “older adults” OR “seniors” OR “elderly” (Abstract) = **457,631**  2= (TI=(“transtheoretical model of change” OR “TTM” OR “Stages of change”)) OR AB (“transtheoretical model of change” OR “TTM” OR “Stages of change”) = **5,404**  3= (TI=(“physical activity” OR “walking” OR “walk*” OR “exercise”)) OR AB (“physical activity” OR “walking” OR “walk*” OR “exercise”) = **793,628**  4= #3 AND #2 AND #1 = **53**  5= #3 AND #2 AND #1 and English (Languages) = **53** |
| Cochrane Library (Ovid)  **n= 81** | #1= (“older adults” OR “seniors” OR “elderly”):ti,ab,kw= **77,894**  #2= ( “transtheoretical model of change” OR “TTM” OR “Stages of change”):ti,ab,kw= **1,204**  #3= ( “physical activity” OR “walking” OR “exercise”):ti,ab,kw= **18,271**  #4= #1 and #2 and #3 = **44**  with English language limiter = 8**1** |

S2: Outcomes of included studies

| Authors, Year, Country Study design | Sample size; Sex (m/f); Dropout.  Age mean (SD) or  Age Range (IQR) | Outcome measurement | Baseline scores for PA/self-efficacy: Mean (SD or SE) | Post-intervention scores for PA/self-efficacy: Mean (SD or SE) | Baseline to follow up difference scores for PA/self-efficacy: Mean (SD or SE) |
| --- | --- | --- | --- | --- | --- |
| Basler *et al.,* 2007 [31]  Germany  Parallel RCT | n=170; IG=86 (m/f) (32/54), CG=84 (m/f) (29/55)  IG=75, CG=72; dropout=23  Age mean (SD)=70.3(4.4), IG =70.09(4.19),  CG =70.56(4.55)  Age range: 65-84 years | PA: 7-day activity diary  Self-efficacy: Not assessed | **IG:**  PA (min/day):  Mean (SD)=15.98(21.1)  **CG**:  PA (min/day):  Mean (SD) =14.11(15.5) | **IG:**  PA (min/day):  6-7weeks Mean (SD)= 29.24(14.6)  6 months Mean (SD)= 29.63(24.2)  **CG**:  PA (min/day):  6-7weeks Mean (SD)= 24.7(16.3)  6 months Mean (SD) = 25.3(19.7) | **IG:**  PA (min/day):  Δ 6-7weeks =13.26(-6.5)  Δ 6 months=13.65(9.6)  **CG**:  PA (min/day):  Δ 6-7weeks=10.59(0.8)  Δ 6 months=11.19(4.2) |
| Greaney *et al.,* 2008 [29]  USA  Parallel RCT | n=1274  IG(m/f)= 470(128/342); CG(m/f)= 496(146/348);  dropout= 308  Age mean (SD):  IG=75.2(6.7),  CG=74.7(6.6) | PA: Yale physical activity survey (YPAS).  Self-efficacy: Six-item exercise self-efficacy scale | **IG:**  PA (YPAS score):  Mean (SE)= 46(1.4)  Self-efficacy:  Mean (SE)= 3.41(0.04)  **CG:**  PA (YPAS score):  Mean (SE)= 46(1.3)  Self-efficacy:  Mean (SE)=3.37(0.04) | **IG:**  PA (YPAS score):  12 months Mean (SE)=46(1.2);  24 months Mean (SE)=47(1.3)  Self-efficacy:  12 months Mean (SE)=3.50(0.05);  24 months Mean (SE) =3.52(0.05)  **CG:**  PA (YPAS score):  12 months Mean (SE)=47(1.1);  24 months Mean (SE)= 47(1.2)  Self-efficacy:  12 months Mean (SE)=3.41(0.04);  24 months Mean (SE)=3.41(0.05) | **IG:**  PA (YPAS score):  Δ 12 months=0(-0.2);  Δ 24 months=1(-0.1)  Self-efficacy:  Δ 12 months=0.09(0.01);  Δ 24 months =0.11(0.01)  **CG:**  PA (YPAS score):  Δ 12 months=1(-0.2);  Δ 24 months=1(-0.1)  Self-efficacy:  Δ 12 months = 0.04(0);  Δ 24 months=0.04(0.01) |
| Hsu *et al.,* 2022 [30]  Taiwan  A single-arm clinical trial | n=30;  MPb=15 (m/f) =4/11 MP=13; (m/f) = 3/10  dropout=2  Age mean (SD)=68.8(4.1);  Age range: 66-89 years. | PA:  Physical activity scale for the elderly (PASE)  Self-efficacy:  Self-efficacy for exercise scale | PASE score:  MPb  Mean (SD)= 122.70(56.73)  MP 12  Mean (SD)= 230.17(55.60)  Self-efficacy:  MPb 12  Mean (SD)= 5.52(2.34)  MP 12  Mean (SD)= 8.32(1.49) | PASE score:  MPb 12 weeks  Mean (SD)=208.14(52.29)  MPb 24 weeks  Mean (SD)=177.92(45.39)  MP 12 weeks  Mean (SD)=264.77(93.52),  MP 24 weeks  Mean (SD)=284.27(154.87)  Self-efficacy:  MPb 12 weeks  Mean (SD) =8.06(1.59),  MPb 24 weeks  Mean (SD) =6.84(1.99),  MP 12 weeks  Mean (SD) =8.32(1.49),  MP 24 weeks  Mean (SD) =8.11(1.48) | PASE score:  MPb  Δ 12 weeks = 85.44(-4.44),  MPb  Δ 24weeks= 55.22(-11.34).  MP  Δ 12 weeks= 34.6(37.92),  MP  Δ 24 weeks= 54.1(99.27)  Self-efficacy:  MPb  Δ 12 weeks =2.54(-0.75),  MPb  Δ 24 weeks =1.32(-0.35).  MP  Δ 12 weeks= -0.29(-0.01),  MP  Δ 24 weeks= 0.02(-0.21) |
| SD: standard deviation, SE: standard error, TTM: Transtheoretical model of change, IG: intervention group, CG: control group, PASE: Physical activity scale for the elderly, PA: Physical activity, n: sample size, MP: participants in the maintenance stage, MPb: participants under the maintenance stage, YPAS: Yale physical activity scale. | | | | | |
